# Detection of lineage-reprogramming efficiency of tumor cells in a 3D-printed liver-on-a-chip model

**DOI:** 10.7150/thno.86921

**Published:** 2023-09-04

**Authors:** Zuyan Lu, Xiangwan Miao, Qianqian Song, Huifen Ding, Shiny Amala Priya Rajan, Aleksander Skardal, Konstantinos I. Votanopoulos, Kerong Dai, Weixin Zhao, Baisong Lu, Anthony Atala

**Affiliations:** 1 Department of Orthopaedic Surgery, Shanghai Key Laboratory of Orthopaedic Implants, Shanghai Ninth People's Hospital, Shanghai Jiao Tong University School of Medicine, Shanghai, 200011, China.; 2 Wake Forest Institute for Regenerative Medicine, Wake Forest University Health Sciences, Winston-Salem, NC, USA.; 3 Department of Cancer Biology, Wake Forest School of Medicine, Winston-Salem, NC, USA.; 4 Department of Biomedical Engineering, The Ohio State University, Columbus, OH, USA.; 5 Department of Surgery, Division of Surgical Oncology, Wake Forest Baptist Health, Winston‑Salem, NC, USA.

**Keywords:** Direct reprogramming, Induced hepatocytes, Triple-negative breast cancers, 3D printing, Organ-on-a-chip

## Abstract

**Background:** The liver metastasis accompanied with the loss of liver function is one of the most common complications in patients with triple-negative breast cancers (TNBC). Lineage reprogramming, as a technique direct inducing the functional cell types from one lineage to another lineage without passing through an intermediate pluripotent stage, is promising in changing cell fates and overcoming the limitations of primary cells. However, most reprogramming techniques are derived from human fibroblasts, and whether cancer cells can be reversed into hepatocytes remains elusive.

**Methods:** Herein, we simplify preparation of reprogramming reagents by expressing six transcriptional factors (HNF4A, FOXA2, FOXA3, ATF5, PROX1, and HNF1) from two lentiviral vectors, each expressing three factors. Then the virus was transduced into MDA-MB-231 cells to generated human induced hepatocyte-like cells (hiHeps) and single-cell sequencing was used to analyze the fate for the cells after reprogramming. Furthermore, we constructed a Liver-on-a-chip (LOC) model by bioprinting the Gelatin Methacryloyl hydrogel loaded with hepatocyte extracellular vesicles (GelMA-EV) bioink onto the microfluidic chip to assess the metastasis behavior of the reprogrammed TNBC cells under the 3D liver microenvironment *in vitro*.

**Results:** The combination of the genes *HNF4A*, *FOXA2*, *FOXA3*, *ATF5*, *PROX1* and *HNF1A* could reprogram MDA-MB-231 tumor cells into human-induced hepatocytes (hiHeps), limiting metastasis of these cells. Single-cell sequencing analysis showed that the oncogenes were significantly inhibited while the liver-specific genes were activated after lineage reprogramming. Finally, the constructed LOC model showed that the hepatic phenotypes of the reprogrammed cells could be observed, and the metastasis of embedded cancer cells could be inhibited under the liver microenvironment.

**Conclusion:** Our findings demonstrate that reprogramming could be a promising method to produce hepatocytes and treat TNBC liver metastasis. And the LOC model could intimate the 3D liver microenvironment and assess the behavior of the reprogrammed TNBC cells.

## Introduction

Liver metastases are commonly detected in a range of malignancies including colorectal cancer (30-50%), pancreatic cancer (30-40%), melanoma (10-20%), lung cancer (4-17%) and breast cancer (6-38%). About 50-70% of metastatic breast cancer cases involve the liver metastasis, with a median survival time of 3 to 15 months 1. Triple-negative breast cancers (TNBC), as a typical metastatic cancer, is the most lethal subtype of breast cancer due to its highly aggressive nature and the lack of treatment options 2. Immunotherapy and targeted therapy, as effective strategies, face considerable obstacles for TNBC patient due to the shortage of tumor targets, heterogeneity of cancers, and existence of cancer stem cells (CSCs) 3. Therefore, how to find novel and effective therapeutic treatments while maintaining the liver functions is crucial.

The concept of cellular plasticity recently proposed by Gurdon *et al.* might provide another avenue for TNBC therapy 4. Cellular plasticity enables the cell to convert into cells with a different identity. By expressing lineage-specific genes via lineage reprogramming, tumor cells can recover benign cell characteristics and successfully achieve phenotypic transformation to become therapeutic targets 5. Breast cancer cells have previously been reported to transdifferentiate into post-mitotic and functional adipocytes 6. However, whether this system is also applicable for TNBC cells to transdifferentiate into hepatocytes remains uncertain.

Moreover, the liver microenvironment plays a vital role for TNBC's liver metastasis. The microphysiological structure possess complex and organized 3D microstructures composed of multiple cell layers and ECM components. However, most *in vitro* studies detecting tumor metastasis usually rely on two-dimensional (2D) culture systems 7, which do not necessarily reflect the heterogeneity and intricacy of the liver tissues *in vivo*. 3D printing technology is an additive manufacturing biotechnology to prepare complex 3D liver structures 8,9, which could spatially pattern multiple cell types and small biomaterial volumes in three dimensions, incorporating into a fully functioning hepatic lobule architecture 10. The 3D Liver-on-a-chip (LOC) model is an effective model with continuous perfusion to simulate the critical physiological functions of liver tissues as minimal-level functional units, enabling the study of tumor physiology and pathological functions 11-13. The 3D printing and LOC technologies could regulate oxygen and nutrient distribution inside the matrix to improve the viability and proliferation of cells in the 3D scaffold. As the main loading medium of secretory factors secreted by hepatocytes, extracellular vesicles secreted by hepatocytes (Hep-EVs) are vesicles with a diameter of 40-100 nm in the extracellular environment, containing multiple bioactive proteins, lipids, and nucleic acids of hepatocytes 14,15. Therefore, the LOC model loaded with Hepatocte-EV could be an ideal model for intimating the structures and ingredients of the liver microenvironment. It could also facilitate the study of the roles of the hepatic microenvironment on lineage reprogramming efficiency in TNBC liver metastasis model.

In this study, we aimed to: (1) Transdifferentiation the TNBC with liver metastasis into hepatocytes through lineage reprogramming; (2) Use single-cell sequencing to analyze cells' fate after reprogramming; (3) Use a 3D-printing LOC models to assessing the behavior of the reprogrammed TNBC cells under the liver microenvironment.

## Methods

### Generation of HiHeps

#### Cell Culture

We purchased the MDA-MB-231 (ATCC HTB-26) cell line from the American Type Culture Collection (ATCC; Manassas, VA, USA). Human primary liver-derived hepatocytes (HUCPI, HUM182641, P1) were purchased from TRL/Lonza (New Jersey, USA). MDA-MB-231 cells were maintained in Dulbecco's Modified Eagle Medium (DMEM, Cytiva, USA); reprogrammed cells were maintained in Hepatocyte Culture Medium (HCM, Lonza, USA) at 37°C.

#### Lentiviral-vector Preparation

**Figure 1:**
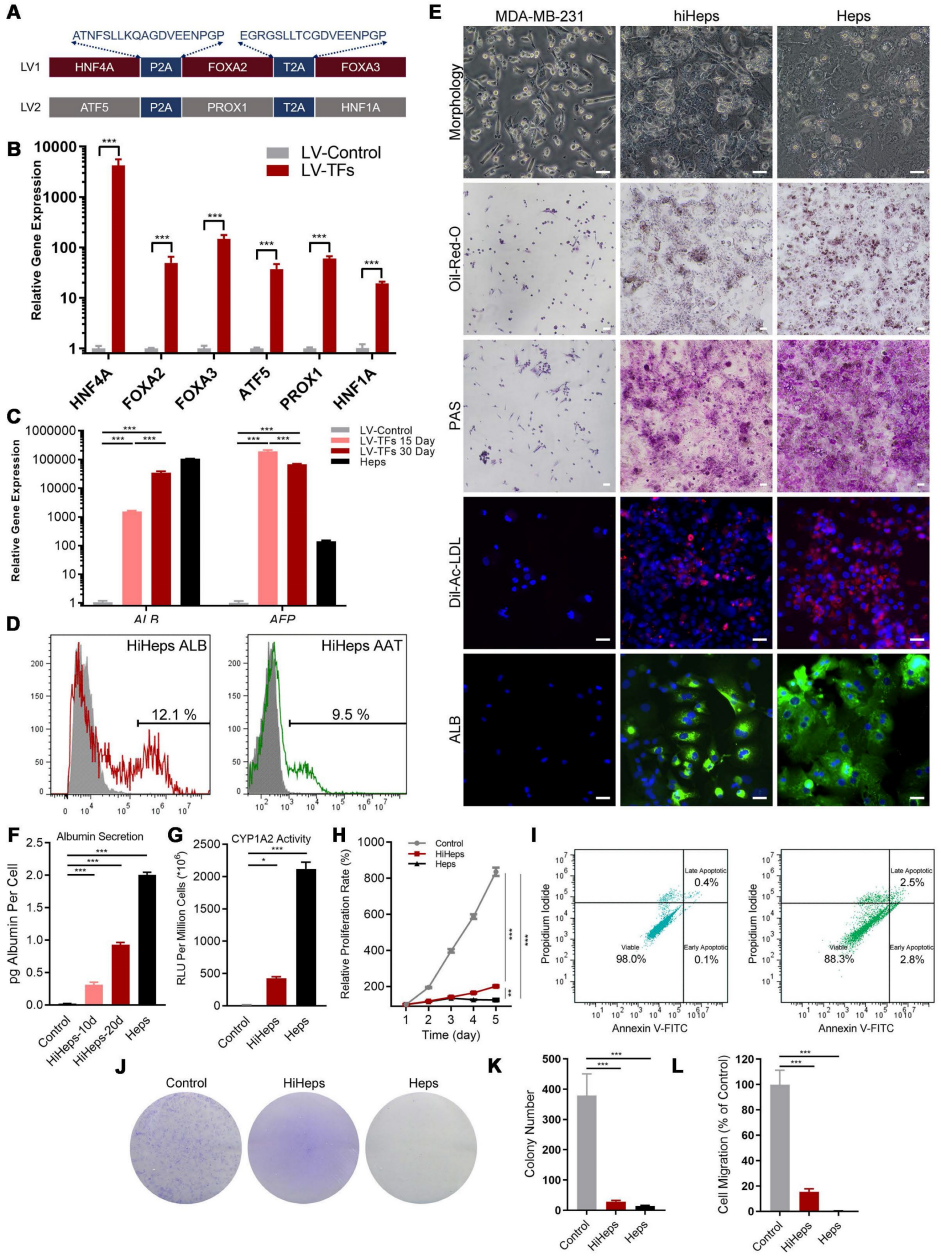
** Generation and characterization of human induced hepatocyte-like cells (hiHeps) reprogrammed from human breast cancer MDA-MB-231 cells. (A)** Hepatic transcription factors expressed from each lentiviral vector. **(B)** Assessing expression of the six transcription factors 2 days after transduction by RT-qPCR (n = 3). **(C)** RT-qPCR analysis of *ALB* and *AFP* expression at days 15 and 30 during reprogramming (Heps: Human primary liver-derived hepatocytes for positive control). Expression levels were normalized to those of MDA-MB-231 cells. **(D)** FCM analysis of *ALB*^+^ and *AAT*^+^ cells 30 days after MDA-MB-231 cells were reprogrammed. **(E)** *In vitro* characterization of fetal and adult hiHeps. Scale bars = 100 μm. **(F)** ELISA results of ALB secretion into culture medium. **(G)** Characterization of drug metabolism-associated enzymatic activities of hiHeps. **(H)** MTT assay of reprogrammed-hiHep viability in hepatocyte medium. **(I)** FCM analysis of cell proliferation/apoptosis after lentiviral transfection. In these results, MDA-MB-231 cells were established as the negative control. Data are presented as means ± SEMs (n = 6). Analysis of variance (ANOVA) was performed for all parameters. Newman-Keuls comparison test was performed after ANOVA. **(J-K)** Colony formation after viral transfection. **(L)** Migration of reprogrammed MDA-MB-231 cells after lentiviral transfection. Data are presented as means ± SEMs (n = 6). Analysis of variance (ANOVA) was performed for all parameters. Newman-Keuls comparison test was performed after ANOVA. The Heps (Hepatocytes) were used as the positive control and MDA-MB-231 cells were used as the negative control.

Two lentiviral-vector plasmids, pLV-hepatocyte nuclear factor 4 alpha (*HNF4A*)-forkhead box protein 2A (*FOXA2*)-*FOXA3* and pLV-activating transcription factor 5(*ATF5*)-Prospero homeobox protein 1 (*PROX1*)-*HNF1A*, expressing six liver-promoting transcription factors (Figure 1A, Table S1), were employed as previously reported 16. We collected lentivirus particles from HEK293T cells transfected with lentiviral-expressing plasmids (pLV-*HNF4A*-*FOXA2*-*FOXA3* or pLV-*ATF5*-*PROX1*-*HNF1A*, 18 µg), the packaging plasmid (psPAX2, 12 µg), and the envelope plasmid (pMD2.G, 6 µg) and then cultured in 15-cm dishes. The lentiviral-vector-enriching supernatant was then concentrated on a KR2i TFF System (Repligen, Waltham, MA, USA) in concentration-diafiltration-concentration mode. Afterward, we determined the viral titer using an Enzyme-linked Immunosorbent Assay (ELISA) Kit for p24 (QuickTiter Lentivirus Titer Kit; Cell Biolabs, Inc., San Diego, CA, USA).

#### Generation of hiHeps

We added concentrated lentiviral vectors pLV-*HNF4A*-*FOXA2*-*FOXA3* (15 ng, p24) and pLV-*ATF5*-*PROX1*-*HNF1A* (45 ng, p24) to 1.0×10^5^ MDA-MB-231 cells grown in 12-well plates (Corning Labware Products, Inc., Corning, NY, USA). Cells were incubated with these lentiviral vectors for 24 h, after which the medium was replaced into HCM.

### Characterization of hiHeps

#### Reverse transcription quantitative polymerase chain reaction

We used an RNeasy Mini Kit (QIAGEN, Germantown, PA, USA) to isolate total ribonucleic acid (RNA) from cells. SYBR Green PCR Master Mix (Thermo Fisher Scientific, Waltham, MA, USA) was used for reverse transcription quantitative polymerase chain reaction (RT-qPCR); primers are listed in Tables S2 and Tables S3. We performed all RT-qPCR experiments on a QuantStudio 3 PCR System (Applied Biosystems). Expression levels were normalized based on glyceraldehyde 3-phosphate dehydrogenase (GAPDH) values. Results are shown as the mean ± standard error of the mean (SEM) of at least three independent measurements.

#### Immunofluorescence

We rinsed the cells three times with phosphate-buffered saline (PBS) before fixing them with 4% paraformaldehyde (PFA) for immunofluorescence (IF) staining. We then permeabilized the cells with 0.3% Triton X-100 and blocked with bovine serum albumin (BSA) (Agilent Technologies, Inc., Santa Clara, CA, USA). Next, we treated the cells with primary antibodies (abs) overnight at 4°C before incubating the cells with the appropriate fluorescence-conjugated secondary ab. Cells were stained with 4′,6-diamidino-2-phenylindole (DAPI) to count their nuclei and then observed under a confocal laser scanning microscope (X-Cite 120 LED Boost; Olympus Corp., Tokyo, Japan).

#### Flow cytometric analyses

For flow cytometry (FCM) analysis, the cells were fixed with 4% PFA and permeabilized with 0.3% Triton X-100 at room temperature (RT). After being blocked with BSA, the cells were incubated with anti-albumin (ALB) ab at 4°C, then they were incubated with the secondary ab in the dark at 4°C, washed twice with Dulbecco's PBS (DPBS), and analyzed on a flow cytometer (Accuri; BD Biosciences, Franklin Lakes, NJ, USA) running FlowJo software (FlowJo LLC, Ashland, OR, USA). Table S4 lists the Antibodies used in IF and FCM analysis.

#### Albumin Enzyme-linked Immunosorbent Assay

We collected the culture media on days 10 and 20 after reprogramming. Medium from primary hepatocytes was collected 1 day after cultivation. We detected ALB using an Albumin ELISA Kit (Alpha Diagnostic Intl. Inc, San Antonio, TX, USA) per manufacturer's instructions.

#### Periodic acid-Schiff staining, Low-density Lipoprotein Uptake, and Oil Red O staining

For Periodic acid-Schiff (PAS) staining, cells were first fixed with 10% formalin before being rinsed three times with distilled water. We then treated cells with Schiff's reagent and washed the cells with ddH_2_O for 5 min. To detect LDL uptake, cultured cells were treated at 37 °C with 1,1′-dioctadecyl-3,3,3′,3′-tetramethylindo-carbocyanine perchlorate (DiI-Ac-LDL; Invitrogen, St. Louis, MO, USA) and then observed under a fluorescence microscope (CKX53, Olympus, Shinjuku-ku, Japan). For Oil Red O (ORO) staining, the cultured cells were fixed with 10% formalin and stained with 60% isopropanol and ORO working solution, followed by two washes with ethanol and four washes with distilled water.

#### Drug Metabolization

For quantitative analysis of cytochrome P450 family 1 subfamily A member 2 (*CYP1A2*) in MDA-MB-231 cells, hiHeps, and hepatocytes (Heps), we added a combination of rifampcin (25 M), 3-methylcholanthrene (3MC; 1 M), and phenobarbital (1 mM) to the medium to activate the cells for 24 h. Then, P450-Glo *CYP1A2* assay reagents (Promega, Fitchburg, WI, USA) were added to the luciferin-ME-containing cell media, and the cells were incubated at 37°C for 1h. We read the luminescence on a Veritas microplate luminometer (Turner Biosystems, Sunnyvale, CA, USA).

#### Colony Formation

For colony formation, monolayer cells were dissociated into single cells and planted in duplicate on six-well culture plates at 1000 cells/well. After 2-3 weeks, cells were stained with gentian violet; after another 2-3 weeks, we counted and evaluated the colonies. The colony formation rate was calculated as colony numbers/planting cells.

#### Transwell *in vitro* Migration Assay

We used a transwell chamber (8-μm pore size; Becton Dickinson, Bedford, MA, USA) for the cell migration experiment. Tumor cells were placed in the filter membrane. After 24 h of incubation at 37 °C, we removed the cells from the upper surface of the membrane using a cotton swab. Cells on the lower surface were fixed with 4% PFA and then stained with 0.5% crystal violet (Sigma Germany, Munich, Germany) for 10 min at RT. We examined the membranes under a microscope (Zeiss Axiovert 200M, Carl Zeiss, Oberkochen, Germany) and counted stained migrated cells in at least five randomly selected fields. Migration potential was then assessed by measuring the number of cells that had migrated to the underside of the transwell membrane.

### Single-cell RNA sequencing

#### Tissue Dissociation for Single-cell RNA Sequencing

Fresh reprogrammed samples were collected by the Tumor Tissue and Pathology Shared Resource (TTPSR) of the Atrium Health Wake Forest Baptist Comprehensive Cancer Center (AHWFB-CCC; Winston-Salem, NC, USA). The cells were put in tissue storage medium (Miltenyi Biotech, Bergisch Gladbach, Germany) at 4 °C, frozen in 10% HybriMax dimethyl sulfoxide (DMSO; Sigma Germany), and preserved in liquid nitrogen for storage. Before sequencing, we thawed and washed the cells in preparation for single-cell RNA sequencing (scRNAseq) using the protocol for human peripheral-blood mononuclear cells (PBMCs; Pleasanton, CA, USA). The Cancer Genomics Shared Resource (CGSR) of the AHWFB-CCC performed all scRNAseq operations. Viable cells (mean, 92.0 ± 4.2%; n = 8) were placed into the wells of a 10×Genomics chromium single-cell capture chip with the goal of a cell recovery rate of 2000-4000 cells. Single-cell gel beads in emulsion (GEMs) were generated on a Chromium Single Cell Controller, and scRNAseq libraries were synthesized using the Chromium Single Cell 3′ Library and Gel Bead Kit per manufacturer's instructions (10× Genomics).

#### Single-cell RNA Sequencing Data Processing

Sample demultiplexing, alignment, filtration, and universal molecular identifier (UMI) count were performed using Cell Ranger single-cell gene expression software (10×Genomics). The obtained data were aggregated for direct comparisons of single-cell transcriptomes. A total of 8062 single cells from two samples were captured, with 3891-4171 cells recovered per channel. Mean reads per cell varied from 111,964 to 120,868, with median Unique Molecular Indexes (UMIs) of 37,709-40,609 per cell. The final cell count was 7672. Next, we used the Seurat toolkit 17 to perform dimension reduction and cell clustering. Data were first loaded into R software 4.0.3 (R Foundation for Statistical Computing, Vienna, Austria) as a count matrix and log-transformed using the NormalizeData function. To remove batch effects, we integrated the count matrices of two samples via IntegrateData, resulting in a batch-corrected expression matrix. Subsequent principal-component analysis (PCA) was performed based on this batch-corrected data. After PCA, we used the first 30 principal components for uniform manifold approximation and projection (UMAP) projection and cell clustering. Cells of the same type were clustered together in the UMAP; cell annotations were then identified based on the expressions of corresponding marker genes.

#### Functional and Pathway Enrichment Analyses

Significantly differentially expressed genes (DEGs) between Transformed Mature Hepatocytes (FMHs) cells (239 DEGs) and Untransformed Proliferating Tumor Cells (UPTs) cells (293 DEGs) were included for functional enrichment analysis in the Gene Ontology (GO) database and pathway enrichment analysis in the Kyoto Encyclopedia of Genes and Genomes (KEGG) database. After obtaining GO annotations and pathway enrichment results for DEGs, we pre-processed the raw data using the ggplot2 and clusterProfiler packages in R version 4.0.3. The calculated *P*-values were Bonferroni-corrected, and a corrected *P*-value of ≤0.05 was set as the threshold. Terms fulfilling this condition were defined as significantly enriched terms in DEGs.

### Preparation and characterization of 3D-printed Liver-on-a-chip (LOC) Model

#### Preparation and characterization of GelMA Bioinks

GelMA was synthesized as described previously 18. We dissolved 8.0-g gelatin (Sigma Germany) in 50 ml of 0.25-M carbonate-bicarbonate buffer at 57°C for 1 h. Then, we added 0.5 ml methacrylic anhydride (MA; Sigma Germany) at 55 °C and stirred the mixture for 3 h. We stopped the reaction by adding 80-ml PBS. We then dialyzed the mixture using 12-14 kDa cutoff dialytic tubes, lyophilized it for 1 week, and stored it at -80 °C until use. We then dialyzed the mixture using 12-14-kDa cutoff dialytic tubes, lyophilized it for 1 week, and stored it at -80°C until use. Next, we measured the methacrylation degree of GelMA using 2,4,6-trinitrobenzenesulfonic acid (TNBS). Different concentrations of GelMA-based solutions were prepared with GelMA, gelatin, glycerol, hyaluronic acid (HA), and a photo-crosslinking agent (Irgacure 2959; Sigma Germany). Prior to the experiments, we mixed 30 mg/mL (3%), 40 mg/mL (4%), or 50 mg/mL (5%) GelMA in DPBS with gelatin (1-3%), 3 mg/mL HA, and 10% (v/v) glycerol; placed the mixture into a tube; and shook it for 3 h in accordance with our previous studies. HA could increase the viscosity of the biolink, while glycerol could ensure smoothness of bioprinting. Afterward, the photo initiator, 20% (v/v) Irgacure 2959 (1% w/v as the stock media), was added to the solutions to form GelMA bioinks. Then, we characterized the flow behavior of the GelMA solution (3-5%) with different concentrations of gelatin (1-3%) using a HR-2 Discovery Rheometer (TA Instruments, Newcastle, DE, USA) with a 25-mm steel parallel-plate geometry. The gap between the plate and the surface of the GelMA solution was 105 μm. Strain was measured at 0.01-100 at an angular frequency of 6.28 rad/s. The rheometer was run with TA Instruments' programmable software, and the temperature of the solution was maintained at 20°C. We collected data for samples in triplicate.

#### Extraction and characterization of hepatocytes extracellular vesicles (Hepatocyte-EV)

Human primary liver-derived hepatocytes (HUCPI, HUM182641, P1) were purchased from TRL/Lonza (New Jersey, USA). Then, P2 hepatocytes were used to extract Hepatocyte -EV. When the cell confluency reached about 70%, the cell culture medium was replaced with serum-free medium. The cell culture supernatant was collected after culturing for 72 h. Cell impurities were removed by centrifugation at 1200 rpm, and then ultracentrifuged. After centrifugation at 100,000 g at 4°C for 90 min, the supernatant was discarded, and the pellet was resuspended in 0.5 mL of DPBS to obtain the Hepatocyte -EV. The concentration of Hepatocyte-EV was detected using a BCA kit (ThermoFisher, MA, USA). The morphology of Hepatocyte-EV was observed using transmission electron microscopy (TEM, TecnaiTM G2 Spirit BIOTWIN). The particle size distribution of Hepatocyte-EV was observed using nanoparticle tracking analysis (NTA, Nanosight NTA v3.2). The effect of Hepatocyte-EV on the proliferation behavior of the MDA-MB-231 cell line was determined using MTT for 2 day and 4 day, respectively.

#### Fabrication of 3D-printed LOC Model

Briefly, the GelMA-Gelatin solution with different concentrations of Hepatocyte-EV and MDA-MB-231 were loaded into the syringe of our Integrated Tissue and Organ Printing (ITOP) system through mechanical mixing by pipette tips for 3D bioprinting. Printing materials were dispensed through 300 µm tapered needles. After lineage reprogramming, GelMA-EV bioinks were injected into the syringe in preparation for tumor construction bioprinting. Throughout the 3D-printing process, we maintained a linear needle speed of 6 mm/s in both the x and y directions, and we used a dispensing pressure of 100 kPa to extrude the bioink solution into microfluidics chips. After manufacture, the structure was immersed in HCM medium, which was changed every 2 days.

#### Accessing cell viability and proliferation

The ultrastructure of the fixed scaffold was examined under a scanning electron microscope (FlexSEM 1000; Hitachi, Tokyo, Japan). Excess water was removed from the samples by freeze drying. We coated all samples with gold using a high-vacuum sputter coater (EM ACE600; Leica, Wetzlar, Germany) before examination. The cell viability were measured inside the 3D-printed scaffold using a live/dead viability kit (Invitrogen). Specifically, samples were treated with 5 μL/mL calcein AM and 2 μL/mL ethidium homodimer for 45 min at 37°C, after which we examined fluorescence using a fluorescence microscope (X-Cite 120 LED Boost; Olympus). Six random fields were chosen for viability analysis using ImageJ software (National Institutes of Health [NIH], Bethesda, MD, USA). Cell proliferation inside the 3D-printed liver tissues was assessed using a 3-[4,5-dimethylthiazol-2-yl]-2,5 diphenyl tetrazolium bromide (MTT) kit. After incubation at 37°C in 5% CO_2_ for 1, 3, 5, and 7 days, we used an MTT Cell Proliferation and Cytotoxicity Assay Kit (Abcam, Cambridge, UK) to determine cell metabolic activity inside the hydrogel scaffold via colorimetric quantification on a multi-mode microplate reader (SpectraMax M5; Molecular Devices, Sunnyvale, CA, USA) at 490 nm.

### Statistics

The GraphPad Prism 9.4.1 (GraphPad Software, Inc., San Diego, CA, USA) and SPSS v9.0 (IBM Corp., Armonk, NY, USA) were used to conduct statistical analyses on functional and histological data. Data are presented as mean ± SEM. Analysis of variance (ANOVA) was performed for all parameters. When an ANOVA revealed significance (*P* < 0.05), we performed a Newman-Keuls test for data analysis.

## Results

### Generation of hiHeps by Direct Lineage Conversion

For hepatic-lineage reprogramming, we used six hepatic transcription factors, *HNF4A*, *FOXA2*, *FOXA3*, *ATF5*, *PROX1*, and *HNF1A*, which proved to generate hiHeps (Figure 1A). Considering the size of the coding sequences for the transcription factors, we chose lentiviruses as the main packaging carriers for their higher loading and transduction efficiencies 19. Then, we validated the efficiency of the integrated liver transduction plasmid (Figure S1). Besides, we further compared reprogramming efficiency among multiple liver metastatic tumor cell lines (SW480: human colon adenocarcinoma cell line; A549: human pulmonary adenocarcinoma cell line; PANC-1: human pancreatic carcinoma cell line; MDA-MB-231: human breast adenocarcinoma cell line). By detecting the mature hepatocytic marker (ALB, Albumin) through PCR, the MDA-MB-231 cell line showed the highest reprogramming efficiency (Figure S2), suggesting that the MDA-MB-231 might have the higher tendency for reprogramming than other liver metastatic cell lines.

### Gene Expression Patterns and Functions of HiHeps resembled Mature Hepatocytes

We first used RT-qPCR to verify that the six transcription factors could be efficiently expressed after transducing MDA-MB-231 cells (Figure 1B). The α-fetoprotein (*AFP*) is an immature hepatic marker whose expression increases during early stage and drops during the end stage of liver reprogramming 20, whereas ALB is a mature hepatocytic marker. We detected *ALB* gene expression 30 days after transduction, and observed a progressive reduction in *AFP* expression, indicating that reprogramming was effective (Figure 1C). FCM analysis found that 12.1±1.1% of cells were *ALB*^+^ and 9.5±0.9 % were positive for Z variant α-1 antitrypsin (*zAAT*) 30 days after reprogramming (Figure 1D). We next verified hiHeps formation and human hepatocytic functional features via PAS, LDL uptake, and ORO staining (Figure 1E). We further confirmed hepatocytic function by successful detection of ALB secretion by ELISA (Figure 1F) and C*YP1A2* activity by fluorescence (Figure 1G). Cell proliferation curves also showed that the proliferative behavior of cells was like that of hepatocytes (Figure 1H), while FCM analysis showed no differences in cell proliferation/apoptosis (Figure 1I). In addition, colony formation results showed that lentiviral treatment significantly inhibited the proliferation and migration of the reprogrammed MDA-MB-231 cells (Figure 1J-L). Taken together, these results were consistent with successful induction of hiHeps with typical hepatic functionals.

### Single-cell Analyses Showed that reprogrammed MDA-MB-231 Cells Showed More Hepatic and Less Metastatic Features

**Figure 2:**
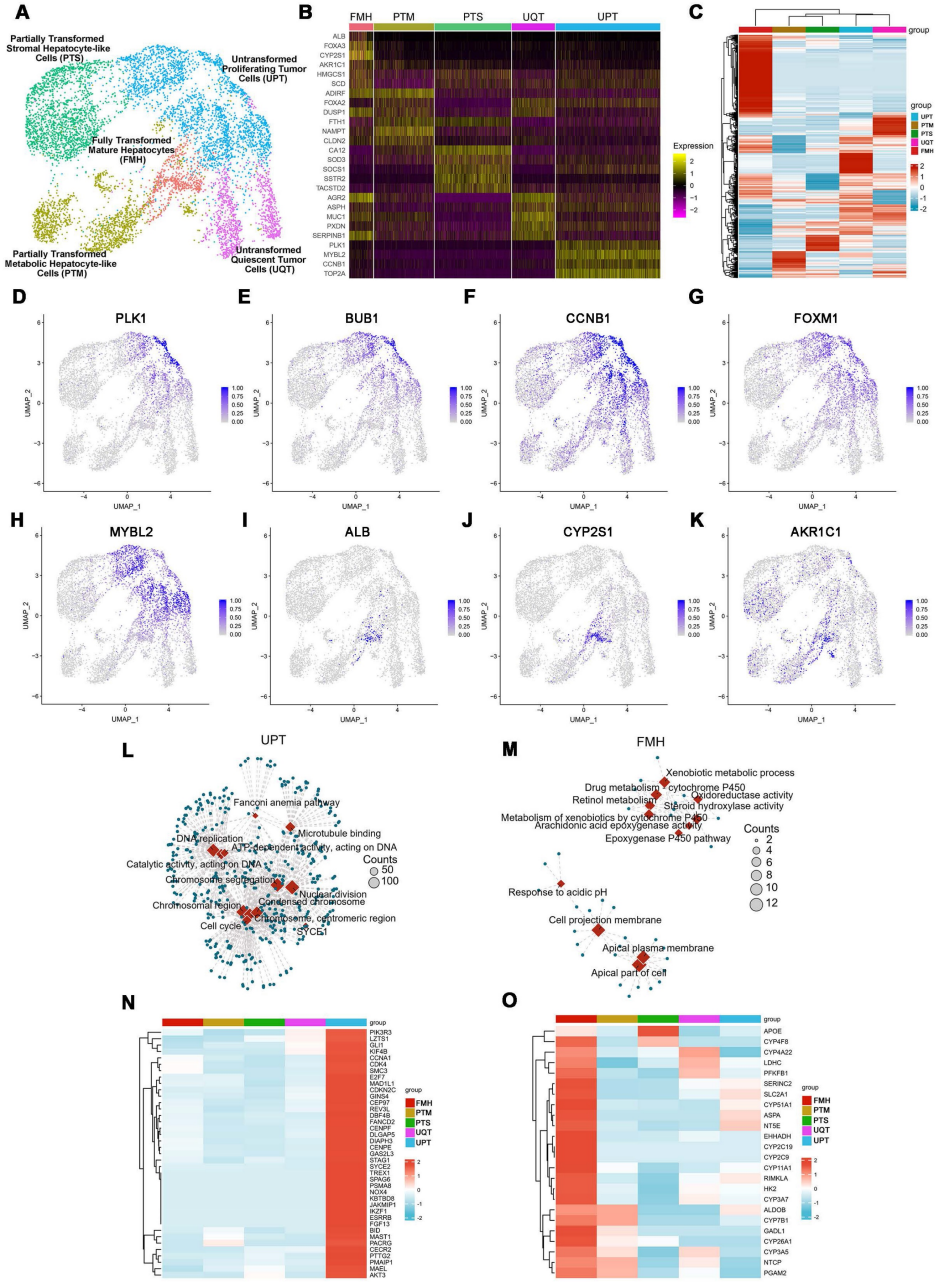
** Characterization of cell fates by scRNAseq after lineage reprogramming. (A)** A *t*-distributed stochastic neighbor embedding (tSNE) plot of scRNAseq data analyzed using Seurat. Cluster numbers, names, and examples of enriched genes are displayed. FMH, fully transformed mature hepatocytes; PTM, partially transformed metabolic hepatocyte-like cells; PTS, partially transformed stromal hepatocyte-like cells; UPT, untransformed proliferation tumor cells; UQT, untransformed quiescent tumor cells. **(B, C)** Heatmap shows expression of DEGs in the five cell subsets. Color scheme is based on *z*-score distribution, from -2 (purple) to 2 (yellow). Genes (rows) with (log2 fold change [FC]) > 1 and adjusted *P* < 0.01 are listed in each subset. The tSNE visualization of gene expression of PLK1 **(D)**, BUB1**(E)**, CCNB1**(F)**, FOXM1**(G)**, MYBL2**(H)**, ALB**(I)**, CYP2S1**(J)**, AKR1C1**(K)** for all clusters that underwent lineage reprogramming. **(L)**. The network diagram for GO and KEGG pathway enrichment analysis of genes in UPT and FMH **(M)**. The color bar at the right indicates gene expression in log2 scale. Red and blue colors represent higher and lower gene expression levels, respectively. **(N)** Expression profiling of genes involved in tumor proliferation-related genes. **(O)** Expression profiling of genes involved in liver metabolism-related genes.

ScRNAseq analysis identified five distinct clusters in reprogrammed MDA-MB-231 cells (Figure 2A-C): Fully Transformed Mature Hepatocytes (FMH), Partially Transformed Metabolic Hepatocyte-like Cells (PTM), Partially Transformed Stromal Hepatocyte-like Cells (PTS), Untransformed Quiescent Tumor Cells (UQT), and Untransformed Proliferating Tumor Cells (UPT).

From the expression patterns of these genes, we observed that lineage-reprogramming treatment decreased the expression of tumor proliferation-associated genes such as *CCND1*, *CCNE1*, *E2F1*, *FOXM1*, *MYBL2*, *TOP2A*, *BUB1*, and *PLK1* 21,22 (Figure 2B, D-H), and increased the expression of genes associated with hepatocytic fate (*ALB*, *ACCS2*, *AKR1C1*, *HMGCS1*, and *SCD*) 23,24 (Figure 2B, I-K). Next, GO analysis and KEGG pathways analysis of upregulated genes in UPT, UQT, PTS, FMH and PTM clusters revealed that the original untreated UPTs shared many characteristics with malignant tumors (Figure 2L-O, Figure S3), such as cell proliferation (nuclear division, mitotic nuclear division, organelle fission) and cell cycle progression (cell cycle) (Figure 2L, N). In contrast, the FMH cluster showed more genes related to hepatocytic metabolism of alcohol, sterol, and toxins (alcohol metabolic process, sterol biosynthetic process) (Figure 2M, O).

High- and low-grade untransformed cancer cells (respectively, UPT and UQT) showed different expression levels of tumor cell markers. UPTs showed high expression of *PLK1*, *MYBL2*, *CCNB1*, and *TOP2A* (Figure 2B, D-H), which are typical markers of MDA-MBA-231 in its proliferation/cell cycle 25. UQTs did not express such high levels of those genes but did express certain tumor cell markers such as *AGR2*, mucin 1 (*MUC1*), and *SERPINB1* 26 (Figure 2B).

Both FMH and PTM showed enrichment in hepatic genes such as ALDOB, CYP7B1, GADL1, CYP26A1, CYP3A5, NTCP and PGAM2 (Figure 2I-K, M, O). However, the FMH cluster expressed higher levels of secretion related gene (*ALB*, *AAT*, *ACSS2*, *HMGCS1*, *SCD, EHHADH, HK2*) 27 (Figure 2B) and drug metabolic relate CYP450 gene (CYP2C9, CYP2C19, CYP3A7) 28 (Figure 2B, O, Figure S3), whereas PTM clusters were mainly enriched in cytokine production and hormone metabolic genes such as *PCSK1N, CLU, AIRE and OSM* 29 (Figure S3). These results suggested that FMH and PTM were likely to behave in a similar hepatic metabolic genetic manner and share similar reprogramming outcomes (Figure 2M, O). Interestingly, PTS showed higher expression of ECM or cell adhesion related genes (FN1, HSD3B1, COL6A2, COL18A1, MGP and THBS1) 30, which suggested that this cluster resembled the hepatic stroma cells in connective liver tissue and vessel, mediating cell-to-cell and cell-to-matrix interactions, heparin binding-related functions (Figure S3). Taken together, these results showed that MDA-MB-231 cells showed more hepatic cell types and fewer metastatic characteristics after lineage reprogramming.

### Preparation and biological characterization of GelMA-Gelatin Hydrogel for 3D Printing

The rheological characteristics of a GelMA solution are important for estimating bioink printability (Figure S4A, B). Therefore, we tested different concentrations of GelMA to analyze their rheological properties. The ideal concentration for 3D-printing material should be self-supporting, since the viscoelastic solid can be extruded as viscoelastic fluid through the nozzle under shear stress 31. Figure S4C shows that at 4-6%, the GelMA solution performed as a self-supportive viscoelastic solid, consistent with the results of our previous studies 18. The addition of the gelatin and ultraviolet (UV) irradiation could dramatically improve GelMA's mechanical properties such as viscosity and shear stress (Figure S4D-E). An important indicator, “flow point” is the intersection of G′ and G″ to demonstrate a hydrogel's flow properties under stress (Figure S4C). Figure S4C shows that concentrations of GelMA solution > 4% exhibited the solid-liquid transition (flow point) under shear stress through the nozzle, which is necessary for the printing process. Moreover, Figure S4D shows that the slope of the flow behavior curves gradually increased with the addition of gelatin, indicating that gelatin could dramatically increase the viscosity of the GelMA hydrogel for 3D printing. Figure S4E shows that the hydrogel's density and shear stress increased after UV irradiation. Taken together, these results indicated that GelMA bioink at 4-6% was the ideal printing material due to its rheological properties before 3D printing.

Next, we used the 3D bioprinter to construct the 3D-printed GelMA-Gelatin hydrogel to simulate liver microstructure (Figure S4F). Figure S4G-H demonstrates that addition of the gelatin could dramatically improve the surface topography of GelMA solution, which was consistent with the results shown in Figure S4C-D. Then, we observed pneumatic pressure (in kPa) for dispensing through the nozzle and calculated that the associated shear stress would increase proportionally with increased concentrations of GelMA and gelatin for testing (Figure S4I). Based on our previous research 18, pneumatic pressure < 150 kPa and shear stress < 2 kPa led to higher cell viability for 3D printing; therefore, we chose a 5% GelMA solution with 2% gelatin for 3D-printing bioink, which offered better printing molding effect, lower printing damage, and higher cytocompatibility (Figure S4J).

### The 3D-printed Liver-on-a-chip (LOC) made of GelMA-EV bioink could support the viability and characteristic of hepatocytes

**Figure 3:**
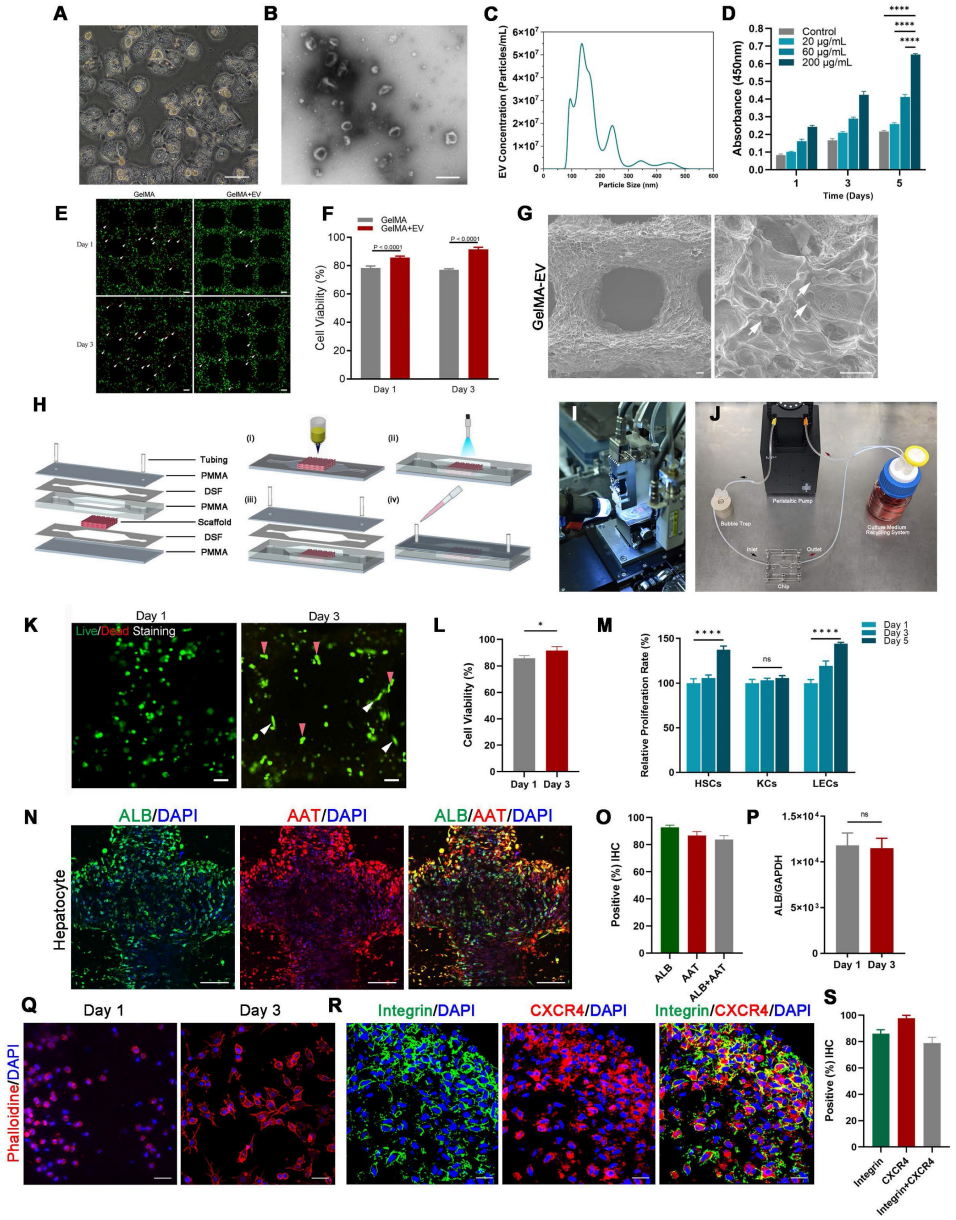
** Preparation and characterization of the 3D-printed Liver-on-a-chip (LOC) model. (A)** The morphology of Hepatocyte **(B)** The TEM topography of Hepatocyte-EV. **(C)** Particle size distribution of Hepatocyte-EV through NTA. **(D)** Hepatocyte-EV promote the proliferation and migration of MDA-MB-231 cells. MTT assay detected the proliferation effect of different concentrations of Hepatocyte-EV under 20 μg/mL, 60 μg/mL and 200 μg/mL (n=4). **(E)** Live/dead staining and semi-quantification **(F)** of MDA-MB-231 Cell viability in 3D-bioprinted GelMA-EV constructs (days 1 and 3). **(G)** SEM images of the 3D printed GelMA-EV scaffold. **(H)** Schematic illustration of the preparation of the model on a 3D printer. **(I)** Fabrication of LOC model by 3D printing. **(J)** Experimental setup of the LOC model. **(K)** Live/dead staining and semi-quantitation **(L)** of the hepatocytes viability inside the LOC at day 1 and day 3. **(M)** Quantitative comparison of cell proliferation (HSCs: hepatic stellate cells, KCs: Kupffer cells, LECs: liver sinusoidal endothelial) for the GelMA-EV bioink through MTT assay. **(N)**. Immunofluorescent detection and quantification **(O)** of the expression levels of ALB and AAT inside the 3D-printed LOC model. **(P)** RT-qPCR detection of expression levels of ALB in the hepatocytes inside the LOC. **(Q)** Cell attachement of the MDA-MB-231 cells inside the LOC. **(R)** IF detection and quantification **(S)** of typical markers (integrin: green; *CXCR4*: red) of MDA-MB-231 cells inside the LOC model. Scale bars = 10 μm. Data are presented as means ± SEMs (n = 6). Analysis of variance (ANOVA) was performed for all parameters. Newman-Keuls comparison test was performed after ANOVA. *, **, *** and **** indicate p < 0.05, p < 0.01, p<0.001 and p < 0.0001 respectively.

Furthermore, to better recapitulate aspects of the human physiologic liver microenvironment, we used the GelMA-Gelatin Hydrogel mixed with the hepatocyte-EV as GelMA-EV bioink to construct a LOC model, based on our previous research 32,33. After characterizing the morphology characteristics of the hepatocytes (Figure 3A), the hepatocyte-EV were collected, and the concentration was detected to 1.04 ± 0.22 mg/mL. TEM showed that hepatocytes extracellular vesicles (Hepatocyte-EV) were in round-shaped vesicular morphology (Figure 3B). The size distribution detected by NTA showed that the average diameter of the hepatocyte-EV was 148.3 ± 5.7 nm (Figure 3C). We then mixed different concentration of hepatocyte-EV (20 μg/mL, 60 μg/mL, and 200 μg/mL) with the GelMA hydrogel and found that 200 μg/mL hepatocyte-EV promoted MDA-MB-231 cell growth the most (Figure 3D). Cell viability results further verified that adding hepatocyte-EV could dramatically improve the proliferation of the MDA-MB-231 cells (Figure 3E, F) and the distribution of the hepatocyte-EV was verified under the scanning electron microscope (SEM) (Figure 3G), which might have facilitated cell attachment inside.

After verifying the printability and biocompatibility of the liver tissues, we then constructed the LOC model with the microfluidic platform to simulate circulatory system inside the liver tissues (Figure 3H-J). The viability for the hepatocytes showed that LOC could maintain the cell viability at 90.09 ± 0.59% at day 3 (Figure 3K-L). We further verified that LOC could maintain the hepatic stellate cells, Kupffer cells, and the liver sinusoidal endothelial cells (Figure 3M) and the expression of ALB and AAT in hepatocytes (Figure 3N-P). Apart from the hepatocyte, the LOC system could also support the proliferation and attachment for MDA-MB-231 cells (Figure 3Q). Staining for integrin and chemokine (C-X3-C motif) receptor (*CXCR4*; a tumor proliferation and invasion marker) also revealed that the LOC could support MDA-MB-231 cell attachment and migration inside the scaffold (Figure 3R, S).

### 3D-printed Liver-on-a-chip Model Detected More liver characteristics and Less Metastasis Behavior of MDA-MB-231 Cells after Lineage Reprogramming

**Figure 4:**
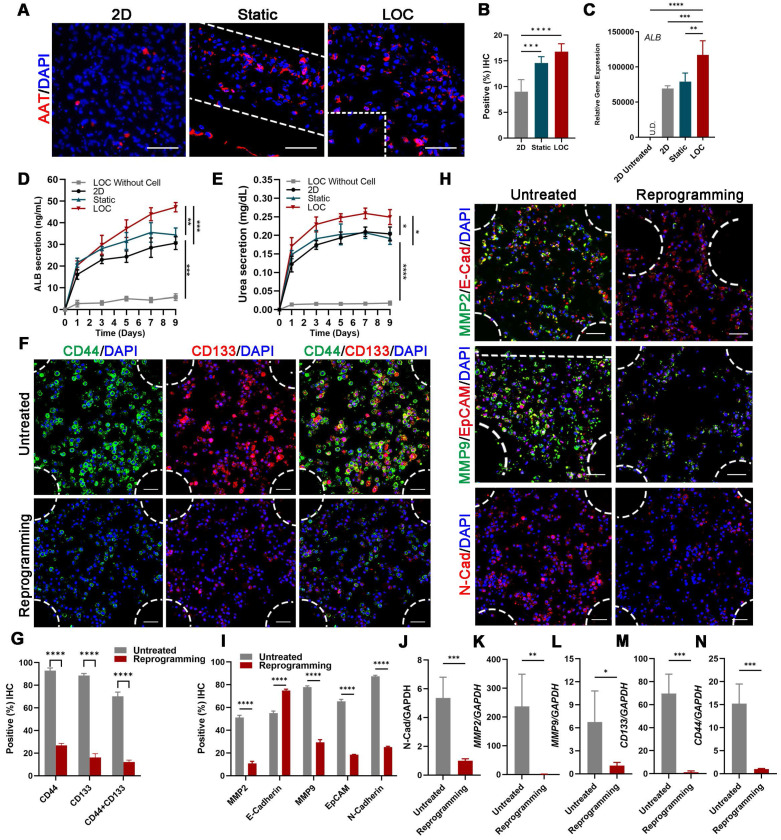
** The characterization of the MDA-MB-231 cells after lineage-reprogramming inside Liver-on-a-chip (LOC) model. (A)** IF detection and quantification **(B)** of hepatic markers (*AAT*: red) of reprogramming cells inside the 2D model, Static and LOC system at day 9. **(C)** RT-qPCR detection of ALB expression at day 9 in 2D model without reprogramming (untreated), 2D model with reprogramming, Static and LOC system. **(D)** Elisa results of ALB and Urea **(E)** secretion of the reprogrammed MDA-MB-231 cells inside the LOC without cells, 2D model, Static and LOC system. **(F, G)** IF detection and quantification of *CD44* and *CD133* expression levels inside the LOC model. **(H, I)** IF detection and quantification of *MMP-2*, E-cadherin, *MMP-9*, *EpCAM*, and N-cadherin expression levels for the reprogramming hiHeps (Reprogramming) and MDA-MB-231 cells (Untreated) in the LOC model. The metastasis-related gene expression of *N-Cadherin* **(J)**, *MMP2* **(K)**, *MMP9* **(L)**, *CD133* **(M)** and *CD44* **(N)** in MDA-MB-231 cells inside the LOC model. Data are presented as means ± SEMs (n = 6). Analysis of variance (ANOVA) was performed for all parameters. Newman-Keuls comparison test was performed after ANOVA. *, **, *** and **** indicate p < 0.05, p < 0.01, p < 0.001 and p < 0.0001 respectively.

Confocal imaging of ALB and AAT staining showed that reprogrammed hepatocyte cells in the LOC were homogeneously dispersed in the gel regions and the AAT positive cells improved from 14.60 ± 1.21% for static to 16.78 ± 1.54% for LOC model, which is much higher than the 2D model with 9.00 ± 2.36% (Figure 4A, B). And the gene expression of ALB (Figure 4C), the Elisa detection of ALB secretion (Figure 4D) and Urea secretion (Figure 4E) secretion revealed similar trends. These results showed that the LOC model could restored the phenotype of newly generated hepatocytes, suggesting reprogramming as a potential method for the production of hepatocytes.

After verify the liver characteristics, we detected cell metastatic behavior. In the reprogramming group, expression levels of cancer stem cell markers *CD44* and *CD133* significantly decreased, from 92.92% to 26.81% and from 88.51% to 16.22%, respectively; moreover, counts of *CD44*^+^ and *CD133*^+^ cells decreased from 70.20% to 12.20% (Figure 4F, G). Relative cancer invasion markers matrix metalloproteinase 2 (*MMP-2*), E-cadherin, *MMP-9*, epithelial-cell adhesion molecule (*EpCAM*), and N-cadherin in the LOC model were detected via immunohistochemical (IHC) staining. The results showed that reprogramming maintained the expression levels of E-cadherin while dramatically decreasing those of the other four markers (Figure 4H, I). The Gene expressions of *N-cadherin*, *MMP2*, *MMP9*, *CD133* and *CD44* further confirmed that the aggressive and metastasis characteristics after reprogramming were dramatically reduced in the LOC system (Figure 4J-N).

## Discussion

The liver is one of the most common organs for TNBC metastasizes, which is associated with the worst prognosis and survival. Approximately 50% of patients with metastatic breast cancer are diagnosed with liver metastasis, and patient will suffer deterioration of liver function due to aggravation of breast cancer liver metastasis (BCLM) 34. Until now, the typical treatment for BCLM refers to chemotherapy, immunotherapy, radiotherapy, and surgery. Despite advances in diagnosis, surgical treatment, and chemotherapy for metastasis, the survival rate for TNBC remains disappointing. Therefore, how to find novel and effective therapeutic treatments while maintaining the liver functions is crucial. In this study, by expressing lineage-specific genes via lineage reprogramming, we presented an alternative treatment for breast cancer liver metastases and proved that TNBC could successfully achieve phenotypic transformation into hepatocytes and inhibit the proliferative behavior of TNBC. Besides, we improved the infection efficiency from 125,000 virus particles/cell 35 to 3000 total lentiviral particles/cell for effective reprogramming using our multi-virus infection system. However, the lineage reprogramming still faces several challenges for its therapeutic applications, such as epigenetic hurdles 36,37 and repressive chromatin states, which still comprise the major mechanistic barriers for epigenetic reprogramming 38. Future work should focus on improving reprogramming efficiency and combing with present therapy to achieve comprehensive treatment for TNBC.

Apart from the tumor cell itself, the hepatic microenvironment, the liver sinusoidal structure and the blood perfusion have also been regarded as the crucial factors for the initial arrest and progression of breast cancer within the liver. And the typical “seed and soil” hypothesis emphasis the interaction between the metastatic breast cancer cells (seed) and the liver microenvironment (soil). However, the conventional method for studying tumor cell proliferation and migration is only restricted to 2D monolayer culture, which cannot replicate the characteristics of the liver microenvironment 7. Animal models are also plagued by dissimilarity to human tissues and high overall expense 39. The three-dimensional (3D) cellular microenvironment play an important role in maintenance of hepatocyte function with superior cell-cell and cell-matrix interactions. However, the traditonal 3D culture model, such as sandwich cultures and spheroid models, are plagued by insufficient oxygen and nutrients distribution inside 31. These shortcomings are mitigated by integrating 3D printing with microfluidic platform to develop LOC platform.

3D printing technology—a novel manufacturing method that uses a layer-by-layer process to build objects—could address the challenges described above by controlling tissue geometry and alleviating poor oxygen distribution inside the scaffold. In liver tissue engineering, the process enables the precise positioning and delivery of hepatocytes and biocompatible ECM-based bioinks, which could precisely mimic the actual liver microenvironments without secondary cell seeding process and facilitate the systematically production of *in vitro* liver models for tissue engineering applications. The LOC chip platform employs a microscale system with continuous perfusion to simulate the critical physiological functions of the liver tissue 40,41. These engineered platforms can control physical factors such as fluid shear stress, concentration, duration, and mechanical deformations of the liver microenvironment with spatial precision 16,32,33. Based on this, the 3D printed liver scaffold can be immobilized within platform microreactors using hydrogel biomaterials, providing a proper microenvironment and allowing for long-term perfusion. This ability to mimic *in vivo* conditions makes these platforms superior to liver static culture systems in plates or single-organoid platforms and to become a good platform for observing tumor cells behavior *in vitro* 42,43. Moreover, the 3D printing porous structure combined with the organ-on-chip model could also control aspects of the oxygen concentration and nutrient distribution inside the liver tissues, which is closer to the liver tissue microenvironment *in vivo*, compared to the 2D culture models 44. Therefore, it is more likely that the 3D printing LOC system could reduce tissue hypoxia and improve nutrition distribution through perfusion, thereby inhibiting cell necrosis inside the tissue and achieving the effect of simulating blood perfusion *in vivo*, according to our previous research 16,31.

Apart from these advantages, the 3D printing liver tissues integrated with LOC system might have great potential in drug development. These platform employs the 3D structure that can be built repeatedly through 3D printing 45. It also employs the microscale system with continuous perfusion to simulate the critical physiological functions of the organ or tissue as minimal level functional units. Multiple of these microphysiological systems can also be integrated to capture the multi-tissue interactions through perfusion 33. We believe that these platforms could be used to observe critical outcomes like drug-induced toxicities on targeted disease organ or bystander healthy organs, off-target drug interactions with other organs or even realize the organ-specific 3D tissues in a multi-tissue environment.

## Conclusion

We reported the generation of hiHeps from the MDA-MB-231 cell line via lentiviral expression of *HNF4*, *FOXA2*, *FOXA3*, *ATF5*, *PROX1*, and *HNF1*. The induced hiHeps showed mature hepatocytic characteristics and inhibited metastatic characteristics. We then used the hiHeps together with a GelMA-EV hydrogel to construct 3D-printed LOC system, and further validated the phenotype of newly generated hepatocytes. We observed that the metastasis of the embedded and reprogrammed cancer cells could be inhibited under the liver microenvironment, which suggests that reprogramming could be a promising method for the production of hepatocytes and treatment for TNBC liver metastasis.

## Supplementary Material

Supplementary figures and tables.
