# Supplementary material for: Detection of lineage-reprogramming efficiency of tumor cells in a 3D-printed liver-on-a-chip model
Source: Theranostics. 2023 Sep 4;13(14):4905–18. doi: 10.7150/thno.86921 (PMC10526656; doi:10.7150/thno.86921)
Supplement: Supplementary file 1 — Supplementary figures and tables. [file thnov13p4905s1.pdf]

Supplementary Table S1. Human transcription factors for hepatic conversion of human fibroblasts.

Gene Name	Genebank Number
HNF4A	NM_178849
FOXA2	NM_153675
FOXA3	NM_004497
ATF5	NM_001193646
PROX1	NM_001270616
HNF1A	NM_000545

Supplementary Table S2. Table of primer sequences for the qRT-PCR.

Primer name	Primer Sequences
ALP-F	5'- CTGGTACTCAGACAACGAGATG -3'
ALP-R	5'- GTCAATGTCCCTGATGTTATGC -3'
AFP-F	5'-TGGGACCCGAACTTTCCA-3'
AFP-R	5'-GGCCACATCCAGGACTAGTTTC-3'

Supplementary Table S3. Table of Taqman probes for the qRT-PCR.

Primer name	Supplier	Catalog #
HNF4A	Applied Biosystems	Hs00230853_m1
PROX1	Applied Biosystems	Hs00896293_m1
ATF5	Applied Biosystems	Hs01119208_m1
ALB	Applied Biosystems	Hs00609411_m1
GAPDH	Applied Biosystems	Hs02786624_m1

Supplementary Table S4. Antibodies for immunohistochemistry

Antigen Name	Type	Supplier	Catalog #
CD133	Rabbit	Abcam	ab19898
CD44	Mouse	Abcam	ab6124
CXCR4	Rabbit	Abcam	ab155090
Integrin	Mouse	Abcam	ab30394
MMP2	Rabbit	Abcam	ab92536
MMP9	Rabbit	Abcam	ab76003
N-Cadherin	Mouse	Abcam	ab98952
E-Cadherin	Mouse	CST	14472
EpCAM	Mouse	Abcam	Ab7504
ALB	Goat	Bethyl	A80-129A
AAT	Rabbit	Invitrogen	711079
Alexa Fluor 488 Anti-Goat IgG	Horse	Vector Lab.	DI-3088
Alexa Fluor 594 Anti-Rabbit IgG	Horse	Vector Lab.	DI-1094
Alexa Fluor 488 Anti-Mouse IgG	Horse	Vector Lab.	DI-2488

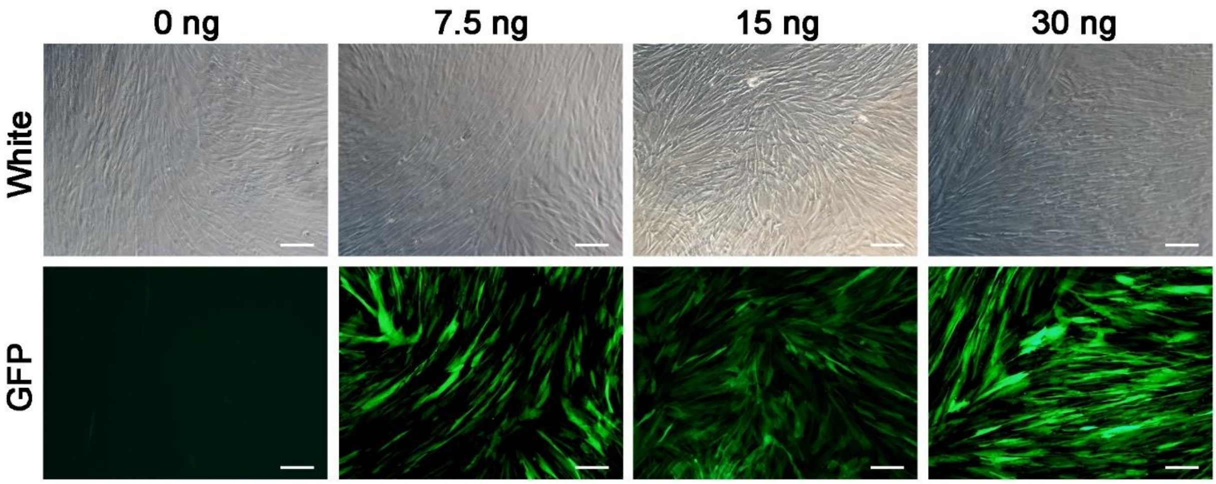

Supplementary Figure 1. Verification of lentivirus-mediated infection system. Each group was transduced with different amount (7.5ng, 15ng and 30ng) of GFP-expressing CmiR0001 Lentivirus for 24 hrs and cultured for 2 days.

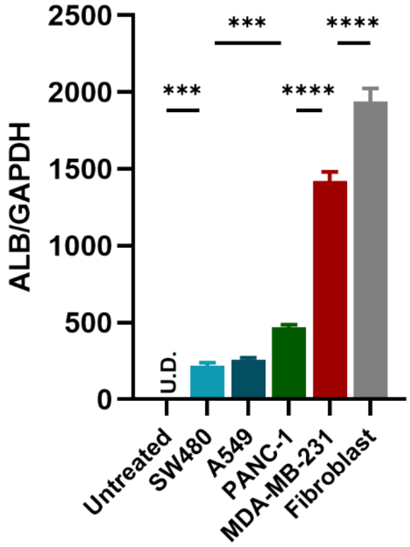

Supplementary Figure 2. RT-qPCR analysis of mature hepatocyte marker (*ALB*) at days 15 during reprogramming. Expression levels were normalized to those of untreated MRC-5 fibroblast cells. SW480: human colon adenocarcinoma cell line; A549: human pulmonary adenocarcinoma cell line; PANC-1: human pancreatic carcinoma cell line; MDA-MB-231: human breast adenocarcinoma cell line. Expression levels were normalized to human untreated fibroblast (as control).

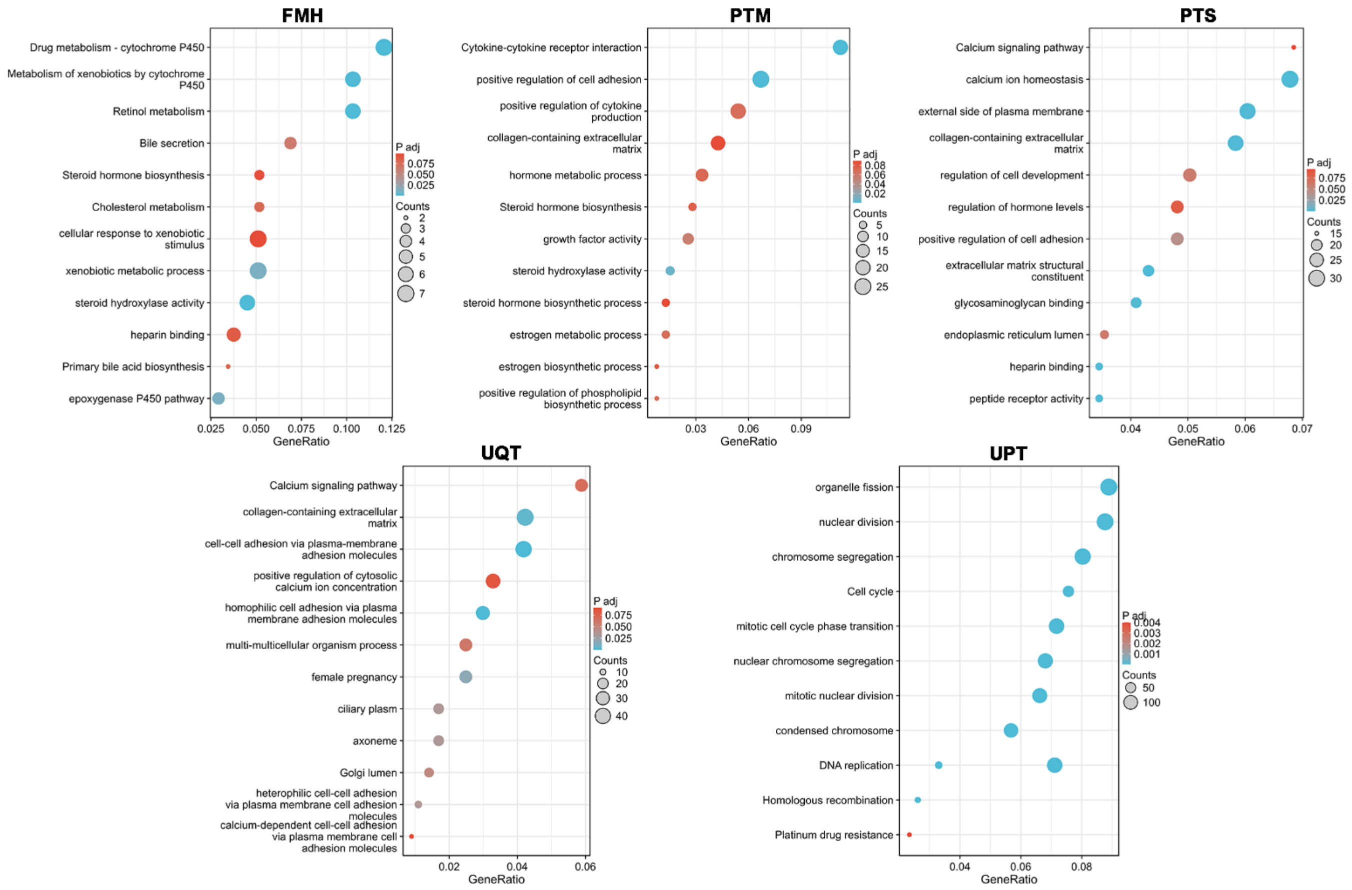

Supplementary Figure 3. The GO functions and KEGG pathways analysis based on the DEGs from the FMH, PTM, PTS, UQT and UPT.

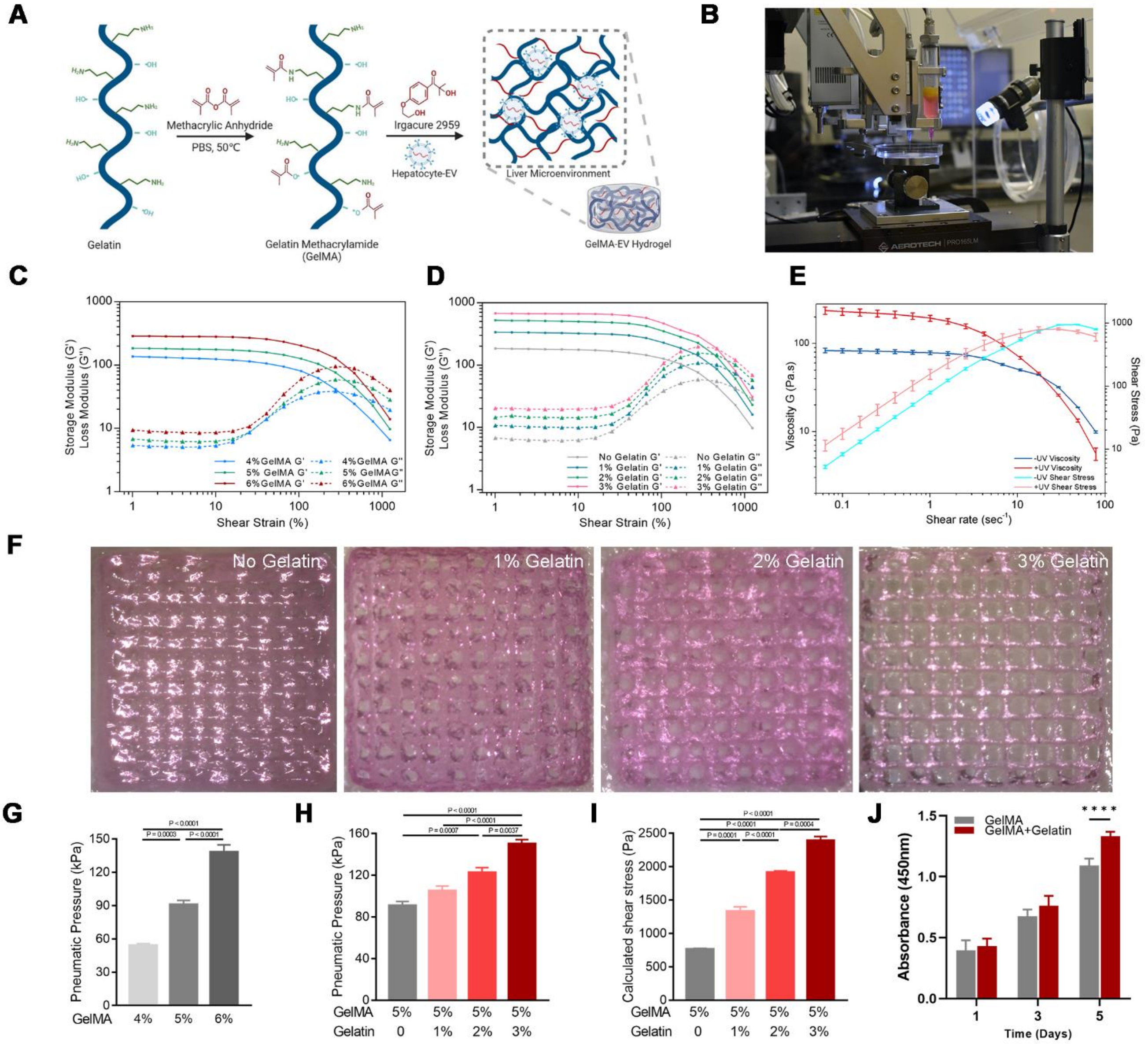

Supplementary Figure 4. Construction and characterization of the 3D-printed GelMA-EV model for simulating liver microenvironment. (A) Schematic diagram of the gelation processes of the 3D-printed GelMA scaffold. **(B)** Fabrication of the 3D GelMA scaffold through 3D printing. **(C)** Dependence of storage modulus (G′) and loss modulus (G″) on shear strain for GelMA at different concentrations. **(D)** Dependence of shear stress on shear rate curves for GelMA at different concentrations of gelatin at 37°C. **(E)** Dependence of viscosity and shear stress on shear rate (sec^{-1}) for 5% GelMA with 3% gelatin before and after UV irradiation at 37°C. **(F)** Top view of the printed scaffold with different concentrations of gelatin. The bioprinted liver scaffold composed of different concentrations of gelatin was fabricated up to 10×10×10 mm in size. **(G)** Printing pneumatic-pressure assessment of four concentrations of GelMA. **(H)** Printing pneumatic-pressure assessment of 5% of GelMA with different concentrations of gelatin. **(I)** Calculated shear stress on different concentrations of gelatin. (**J**) Cell proliferation was tested via MTT assay on days 1, 3, and 5. Data are presented as means ± SEMs (n = 6). Analysis of variance (ANOVA) was performed for all parameters. Newman-Keuls comparison test was performed after ANOVA. *, **, *** and **** indicate $p < 0.05$, $p < 0.01$, $p<0.001$ and $p < 0.0001$ respectively.
